# Mentalizing on the edge: borderline traits and their association with real-time self-reflection - an ecological momentary assessment study

**DOI:** 10.1186/s40479-026-00347-1

**Published:** 2026-05-12

**Authors:** Inken Höller, Sarah Schwitzky, Thomas Forkmann, Saskia Forster, Philip S. Santangelo

**Affiliations:** 1 Department of Clinical Psychology and Psychotherapy, University of Duisburg- Essen, Universitätsstr. 2, 45141 Essen, Germany; 2 Institure for Mental Health and Behavioral Medicine, Department of Psychology, Health and Medical University Düsseldorf/Krefeld, Düsseldorf/Krefeld, Germany; 3 Institute of Medical Psychology and Medical Sociology, RWTH Aachen University, 52074 Aachen, Germany; 4 Department of Behavioural and Cognitive Sciences, Université du Luxembourg, Esch-sur-Alzette, L- 4366 Luxembourg

**Keywords:** Mentalizing, BPD, Ecological momentary assessment

## Abstract

**Background:**

Mentalization plays a key role in borderline personality disorder (BPD), yet its moment-to-moment fluctuations remain understudied. This study is the first to use established self-report items within an Ecological Momentary Assessment (EMA) to explore the links between aspects of mentalizing in daily life and BPD traits in a community sample.

**Methods:**

The non-clinical sample consisted of 86 participants (86% female) aged between 18 and 51 years (*M* = 22.93, *SD* = 5.36). As part of a baseline survey in the laboratory, the participants completed self-report questionnaires to assess borderline accentuation and mentalizing in the form of subjective certainty regarding their own and others’ mental states (self-certainty and other-certainty). In addition, in a subsequent one-week EMA, they reported momentary self-certainty and other-certainty at six randomized times during the day.

**Results:**

Using multiple regression (enter method), self-certainty proved to be a negative predictor of borderline accentuation, with intraindividual variations correlating positively with borderline personality style. Contrary to expectations, however, neither the level of other-certainty in the EMA nor the corresponding intraindividual fluctuations were related to borderline accentuation.

**Conclusion:**

Future studies should consider the high intraindividual variability of self- and other-certainty in order to obtain ecologically valid insights into mentalizing processes in the context of BPD. Promoting self-certainty may be a valuable target in BPD treatment. However, other-certainty might not be as important.

## Background

Borderline personality disorder (BPD) is a severe mental disorder linked to significant impairments in social, occupational, and overall functioning [1, 2]. It often co-occurs with other psychiatric conditions and is associated with reduced well-being and increased life stress [3–5]. Although estimates vary, BPD prevalence in the general population is generally low. Two meta-analyses report rates around 2% [6, 7], with most studies showing rates near 1% in general Western populations [e.g., 8, 9], but over 3% among young adults [e.g., 10, 11].

Given the high impairment and prevalence of BPD, understanding its development and maintaining factors is crucial for effective treatment. Mentalization-based therapy (MBT), developed by Bateman and Fonagy [12], is a well-established approach with a strong empirical foundation [13]. MBT targets patients’ capacity to understand their own and others’ mental states – a capacity often disrupted in BPD. Impaired mentalizing, particularly in interpersonal contexts, is considered central to the disorder’s development and pathology [12, 14, 15].

Impaired mentalizing is closely associated with deficits in areas such as attentional control of mental states, emotion regulation, self-control and the coherent organization of the self [15–21]. These processes – especially together with the distorted perception of others’ emotions, intentions or actions – could contribute to individuals with BPD being overwhelmed by intense affect, experiencing an unstable sense of self, having high reactivity in social situations, being more sensitive to social rejection and showing inappropriate responses to interpersonal stress. This in turn could explain the impulsive reactions and unstable relationships typical of BPD [15, 22–24].

Although research on mentalizing has increased significantly in recent decades [25] and specific connections between mentalizing and BPD are assumed on the basis of empirical findings [15], the majority of studies have disregarded the fact that the mentalizing process is understood as a state that is subject to intra-individual fluctuations [21, 26]. Thus, hardly any studies so far have assessed momentary mentalization in everyday life taking into account temporal dynamics [27].

Overall, few studies have investigated the fluctuations over time of mentalizing in the context of BPD. In some studies, patients’ expressions within psychotherapy sessions were rated by observers with regard to the level of reflective functioning indicating that the mentalizing ability of patients with BPD varies considerably within sessions. In a study by Möller et al. [28], in two sessions of 15 individuals with BPD and comorbid substance abuse disorder, at least 78% of the variation in mentalizing was due to within-person level, i.e. differences between statements within a session. This is in line with a 20-session study of a patient with BPD [29]. Based on the within-session data of 88 patients with BPD undergoing psychotherapy, Kivity et al. [30] attributed moments of better mentalization to lower arousal measured with coder based ratings.

The assessment method ideally suited to examine dynamic processes is Ecological Momentary Assessment (EMA). EMA offers the possibility to repeatedly assess the constructs of interest in the natural environment of participants via smartphones prompting participants to rate their momentary state [31]. Steinberg et al. [27] conducted the first EMA study (*n* = 20, non-clinical sample), which showed that mentalizing can be recorded with high temporal resolution in everyday life. In the baseline survey, the Reflective Functioning Scale [RFS; 32] was applied as an external assessment instrument for mentalizing. In order to capture mentalizing in the current moment in the EMA, the respondents were asked to describe the most emotionally intense moment since the previous measurement time in an audio recording with the smartphone. Trained coders then rated the statements using the RFS. At 67%, a large part of the total variance in mentalizing could be attributed to intraindividual variability. In addition, higher self-reported symptoms of BPD were associated with greater fluctuations in mentalizing from assessment point to assessment point [27]. This provided important insights into intraindividual fluctuations in mentalizing capacity and their relevance in the context of BPD.

Although the RFS is considered the gold standard measurement instrument for mentalizing [33], its applicability in large-scale quantitative studies is questioned due to the high effort and cost involved and the need for well-trained raters [33–36]. In view of the need for further research in the field of EMA, it therefore seems sensible to develop new resource-efficient measurement instruments for smartphone-based research designs that enable the replicability of the findings of Steinberg et al. [27] to be tested in large samples and thus contribute to the empirical basis of the dynamics of mentalization.

However, the RFS only operationalizes certainty and uncertainty on a metacognitive level but not divided into self and others, even though mentalization research has begun to operationalize degrees of certainty about mental states of self versus others as a core feature of mentalizing that is partly decoupled from generic metacognitive confidence [36]. Work on neural representations shows distinct systems for decision uncertainty in metacognition versus mentalizing others or the self [37]. In the meantime, more economical self-report instruments have been developed for the assessment of one’s own mentalization capacity, such as the Certainty About Mental States Questionnaire [CAMSQ; 36]. The CAMSQ uses two scales to assess other-certainty (i.e., the subjective certainty in relation to other people’s mental states) and self-certainty (i.e., the subjective certainty in relation to one’s own mental states) and has shown very good psychometric properties for the English and the German versions [36]. The advantages of the CAMSQ are that it takes into account common definitions of mentalizing by distinguishing between one’s own and other people’s mental states and that its items include various states such as feelings, thoughts, intentions, attitudes, motives, and goals [36]. Additionally, both low and excessively high certainty are treated as maladaptive (hypo- and hypermentalizing [36]). An other-self discrepancy (greater certainty about others than self) is proposed as a hypermentalizing (overinterpreting other people’s thoughts, feelings or intentions) marker and relates to personality pathology [36].

In the frame of a bigger project, we already investigated the psychometric validity and its capacity to reliably track changes over time of a 4-item instrument on the base of the CAMSQ optimized for the use in EMA [38]. We found good psychometric properties and high intraindividual fluctuations of both self- and other-certainty. The subsequent goal of this study is therefore to examine the assumptions of Müller et al. [36] regarding the relationship between psychopathology and self- and other-certainty using borderline characteristics. However, the goal of this study was not to fully cover all aspects of mentalizing and clinical characteristics of BPD but to give a first approach on assessing aspects of mentalizing in EMA and its associations with BPD features in a non-clinical community sample. At this point it cannot be excluded that the items used in this study do not capture mentalizing as a balanced multidimensional construct.

In line with results from Steinberg et al. [27] indicating greater fluctuations in mentalizing in BPD, we expect that the higher the borderline accentuation, the greater the intrapersonal fluctuations in mentalizing in the EMA in our first hypothesis.

Empirical findings also suggest that BPD is associated with lower certainty regarding one’s own mental states [34, e.g., 39]. Furthermore, subjective assessments of mentalizing capacity with respect to one’s own mental states appear to be negatively related to BPD [e.g., 40]. Therefore, the second hypothesis states that the higher a person’s borderline accentuation, the lower their average self-certainty.

Hypothesis 3, in contrast, assumes that higher borderline accentuation is generally associated with higher other-certainty. This hypothesis is primarily based on findings showing a positive relationship between subjective certainty regarding others’ mental states and psychopathology [36], as well as traits of BPD [41, 42].

Finally, based on the positive association between hypermentalizing (overinterpreting) and BPD [e.g., 43, 44], as well as the link between the discrepancy between other- and self-certainty and psychopathology [36], hypothesis 4 posits that higher borderline accentuation is, on average, associated with a greater discrepancy between other- and self-certainty (marker of hypermentalizing).

## Methods

The present investigation was part of a larger study. Further information on the study design and the data set have been published elsewhere [see 38]. The project was preregistered on OSF (DOI 10.17605/OSF.IO/NWG89). Only the aspects of the study design and measurement instruments relevant to the current research question are described below.

### Sample

Based on an a priori power analysis, a sample of at least *N* = 70 was aimed for to detect medium effects with a statistical power of 0.80, a satisfactory average compliance of 75% [cf. 45] and an ICC of 0.40 [cf. 46]. A total of 101 people took part in the study, but the data of 15 participants were excluded from the analyses due to compliance rates below 75%. The power analyses were conducted for multilevel models referring to the main hypotheses of the project.

The resulting sample consisted of *N* = 86 people. The participants were students at the University of Duisburg-Essen and were between 18 and 51 years old. The mean age of the sample was 22.93 years (*SD* = 5.36). Of the 86 participants, 74 (86%) were female. The majority stated that they were currently single (*n* = 81; 94.2%) and speak German as their mother tongue (*n* = 75; 87.2%). A mental illness in the past was reported by 19 participants (22.1%), while 12 (14%) were suffering from a mental illness at the time of the assessment. On average, the sample had a compliance rate of 90.9% in the EMA, which reflects a very high response rate.

### Procedure

The study design is based on the approach of Steinberg et al. [27]. The study consisted of a 30-minute baseline survey in the laboratory and a subsequent smartphone-based assessment in the participant’s everyday lives over seven days. After the subjects gave their informed consent, they completed a computer-based baseline survey via www.soscisurvey.de at the University of Duisburg-Essen. The entire baseline assessment involved questions on socio-demographics as well as various self-report questionnaires on topics such as mentalizing, mood, stress, loneliness, personality, and self-esteem. After completing the baseline questionnaire, the participants received detailed information about the EMA phase. They were asked to answer the same questions several times a day on their smartphone using the app m-Path [47]. They were given instructions on how to install, set up and use the app.

The smartphone prompts started on the day following the baseline assessment. The participants received surveys, consisting of 24 items in total, six times a day at randomized times between 9 am and 9 pm. This resulted in a total of up to 42 measurement points per person. The average interval between two surveys was two hours, with a minimum interval of 30 min. Responses could be postponed by up to half an hour. If a survey had not been completed after 15 min, participants received a reminder. They were instructed to indicate the extent to which the participants agreed with the presented statements at that moment or in relation to the period since the last survey. The items were always presented in the same order. The study was approved by the Ethics Board of the Institute of Psychology of the University of Duisburg-Essen (EA-PSY18/23/07092023) and is in accordance with the Declaration of Helsinki.

### Measures

#### Borderline accentuation

Due to the non-clinical sample, no clinically relevant and diagnostic characteristics of BPD were collected, but instead the degree of a non-pathological borderline accentuation. Therefore, the Spontaneous-Borderline Scale of the German Personality Styles and Disorder Inventory [PSDI; 48] was used in the baseline survey. The PSDI is a self-report instrument for the assessment of non-pathological personality styles, each of which can be assigned to one personality disorder diagnosis. The Spontaneous-Borderline Scale thus captures a non-pathological borderline accentuation that reflects a strong spontaneity of experience and behavior, and intense emotionality. It consists of 10 items (e.g., “I often feel an inner emptiness”). For each question, the respondents were asked to give the answer that was generally most appropriate for them personally and used a four-point Likert scale from 0 = *strongly disagree* to 3 = *fully agree* [48]. An average score was calculated from the 10 items, whereas higher scores indicate a higher borderline accentuation. Regarding satisfactory construct validity, Kuhl and Kazén [48] reported a consistent pattern of correlations with clinical and non-clinical constructs such as other personality inventories, suicidality, and alcohol addiction. In the present study, the internal consistency was very good, with Cronbach’s α = 0.86.

#### Mentalizing

To assess self-reported momentary mentalizing in EMA, four items from the German CAMSQ [36] were selected based on the highest factor loadings. These items were rephrased so that the respondents stated the extent to which they agreed with the statements in relation to the period since the last EMA survey. For self-certainty, the items were “In the period since the last survey, I knew what my virtues are.” and “In the period since the last survey, I knew the reasons for my behavior.” For other-certainty, the items “In the period since the last survey, I recognized when other people did not give their honest opinion.” and “In the period since the last survey, I knew how a person felt when I looked at their face.” were used. A five-point Likert scale from 1 = *not at all* to 5 = *completely* was used to answer the questions. Both scales were evaluated by averaging the two corresponding items. A higher value indicates a greater momentary subjective certainty regarding one’s own or others’ mental states in the period before the respective measurement point [cf. 36]. The aggregation of all 42 measurement points in EMA possibly provides the most accurate estimate of the general mentalizing ability in the seven-day survey period. The Spearman-Brown-correction showed excellent internal consistency for self- and other-certainty (ρ’_SC_ = 0.93; ρ’_OC_ = 0.97). For more information on the scale see Höller et al. [38].

### Statistical analyses

IBM SPSS Statistics Version 28.0 [49] and R Version 4.1.1 [50] were used for the analyses. For the first hypothesis, the MSSD for self-certainty and other-certainty was determined. The MSSD is a measure of intrapersonal fluctuation that considers the temporal order of the measurement points. For each person, the average of the squared differences between consecutive measurement points was calculated [51, 52]. Higher values mean higher fluctuation over time [53]. The relationships between borderline accentuation and the MSSD of self-certainty and other-certainty (hypothesis 1) were tested using Pearson’s *r*.

The relationships between borderline accentuation and mentalizing (hypothesis 2 and 3) were investigated with semi-partial correlations because due to the intercorrelation of self-certainty and other-certainty, the effect of the respective other scale must be statistically controlled for [cf. 36]. Finally, a condition-based regression analysis [CRA; 54] was conducted to investigate whether a greater difference between other-certainty and self-certainty is associated with higher borderline accentuation (hypothesis 4). This method allows to assess discrepancy effects between variables, independently of the separate effects of both variables [36, 54]. Borderline accentuation was first regressed on the two predictors self-certainty and other-certainty in a multiple regression, using the Enter method. Multicollinearity was not given, as all predictor correlations were below *r* =.80 [55], tolerance values exceeded 0.10, and all VIFs were below 10 [56–58]. It was then tested whether the two regression coefficients of the predictors had significantly opposing effects, assuming that other-certainty was a positive and self-certainty a negative predictor of borderline accentuation. A significantly positive auxiliary parameter *abs* (*abs* = |b_OC_ - b_SC_| - |b_OC_ + b_SC_|) with the first part reflecting the magnitude of discrepancy in the effect size of the two predictors and the second part reflecting the magnitude of their combined average effect. Thus, a positive difference in the regression coefficients means that, in accordance with hypothesis 4, higher differences between other-certainty and self-certainty tend to be associated with higher borderline accentuation [36, cf. 54].

For correlation analyses and the t-test, one-sided hypothesis tests were performed. The significance level α was set at 0.05. For the interpretation of the correlations, the guidelines by Cohen [59] were used, according to which values above |*r*| = 0.30 indicate a moderate and above |*r*| = 0.50 a strong correlation. In addition to the described analyses, it was post-hoc decided to test whether there was a significant mean difference between self-certainty and other-certainty, using a *t*-test for dependent samples. Relevant descriptive statistics were also presented. Regarding self-certainty, other-certainty and the other-certainty-self-certainty differences, for each completed EMA survey one difference value and two mean values were calculated per person. All measurements of the respective variable were then averaged by person, and the descriptive statistics were reported for the whole sample.

## Results

### Descriptive statistics

The descriptive statistics are presented in Table 1. Regarding borderline accentuation, the participants had a mean value of 0.97 (range: 2.60), which, on average, corresponds to low agreement with the items. The descriptive statistics also indicated that the participants were on average more confident about their own mental states than about the mental states of others. The paired *t*-test showed that this mean difference between self-certainty and other-certainty was significant and moderate (*t*(85) = 4.93, *p* <.001, *d* = 0.53). Accordingly, the mean difference scores other-certainty — self-certainty were negative (*M* = -0.28). Here, too, the large range from − 1.82 to 1.96 indicated that there were substantial differences between the participants.

**Table 1:** Descriptive statistics

Variable	M	SD	Min	Max	Md	Range
self-certainty	3.91	0.61	2.18	4.99	3.95	2.81
other-certainty	3.64	0.67	1.79	4.98	3.59	3.19
other-certainty-self-certainty-difference	-0.28	0.52	-1.82	1.96	-0.23	3.78
PSDI-Borderline	0.97	0.62	0.10	2.70	0.80	2.60

Note. self-certainty (mean values between 1 and 5), other-certainty (mean values between 1 and 5), difference other-certainty – self-certainty (possible values between − 4 and 4), PSDI-Borderline: Borderline subscale of the Personality Styles and Disorder Inventory to assess borderline accentuation (mean values between 0 and 3), *M*: Mean value, *SD*: Standard deviation, Min: Minimum, Max: Maximum, *Md*: Median

### Fluctuations of momentary mentalizing in EMA

The MSSDs as a measure of intraindividual mentalizing fluctuations at the person level were all > 0, even if some individuals only narrowly exceeded this limit. The MSSD for self-certainty varied between 0.01 and 3.41 and was on average 0.61 (*SD* = 0.56). The MSSD for other-certainty showed a slightly higher mean value of 0.94 (*SD* = 0.83) and a wider range, with a minimum of 0.02 and a maximum of 3.96. Against expectations, there was no significant association between the MSSD of other-certainty and the level of borderline personality style (H1; *r* =.06, *p* =.300). In contrast, the MSSD of self-certainty correlated significantly positively with borderline accentuation (H1; *r* =.26, *p* =.008).

### Relationship between mentalizing and borderline personality style

The zero-order correlation between self-certainty and borderline accentuation was significantly negative and moderate (*r* = − .46, *p* <.001). When statistically controlling for other-certainty, the semi-partial correlation between self-certainty and borderline accentuation was slightly lower, but still significantly negative and moderately strong (H2; *r*_sp_ = − 0.36, *p* =.001). Accordingly, 13% of the total variance of borderline accentuation can be explained by self-certainty alone, when the shared variance with other-certainty is partialized out. In contrast, other-certainty did not correlate with borderline accentuation when statistically controlling for self-certainty (H3; *r*_sp_ = 0.03, *p* =.789). However, without controlling for self-certainty, there was a significantly negative and weak association between other-certainty and borderline accentuation (*r* = − .29, *p* <.001).

The results of the multiple regression analysis in Table 2 are relevant for the examination of discrepancy effects. Self-certainty was a negative predictor of borderline accentuation (*β* = -0.49, *p* <.001), while other-certainty did not predict it (*β* = 0.04, *p* =.786). Regarding hypothesis 4, there was a positive difference in the regression coefficients (*β*_OC_ – *β*_SC_ = 0.53), but the discrepancy effect was not significant (*abs* = 0.08, *p* =.393). This means that a higher borderline accentuation was not significantly associated with a higher discrepancy between other-certainty and self-certainty.

**Table 2:** Standardized regression coefficients of the multiple regression analysis

Predictor	β^a^	SE_β_^a^	z	*p*
self-certainty	-0.49	0.13	-3.68	< 0.001
other-certainty	0.04	0.15	0.27	0.786

Note. Prediction of borderline accentuation by the predictors self-certainty and other-certainty, using the Enter method. *R*^2^ = 0.21. *β*: Standardized regression coefficient, *SE*: Standard error, a: 95% confidence interval of the regression coefficients and standard errors using bias-corrected (BC) bootstrapping with 5,000 samples

## Discussion

The aim of this study was to examine whether the intraindividual fluctuations in mentalizing and their associations with BPD traits identified in an initial EMA study [27] can be replicated using economical self-report items for momentary mentalizing.

Höller et al. [38] already investigated the highly temporal dynamics of mentalizing in this sample. However, additionally, we investigated MSSDs to obtain an additional measure of intraindividual fluctuations in mentalizing at the person level. The MSSDs for self-certainty and other-certainty indicate that participants’ responses varied across consecutive measurement points. Descriptively, intraindividual variability in other-certainty was again higher than that in self-certainty. The intraindividual variability was lower than in the study of Steinberg et al. [27]. A reason could be the diverging measurement approach applied in this study (self-report vs. third-party rating). People might tend to give more consistent answers or choose similar responses when repeatedly surveyed with the same items; still, both self-certainty and other-certainty fluctuate substantially.

The identified MSSDs were used to test the first hypothesis. In line with our first hypothesis, borderline accentuation positively correlated with MSSDs of self-certainty. Though weak, this aligns with Steinberg et al. [27] and suggests that individuals with stronger BPD traits experience greater fluctuations in their subjective certainty about their mental states in daily life. This supports the view of BPD as a disorder characterized by high instability in self-image [60–62]. However, we did not find this correlation for other-certainty. This contradicts the assumptions that, especially within the context of BPD, factors such as emotional arousal or the activation of the attachment system influence the ability to mentalize in interactions with others [15, 63], thereby contributing to intra-individual differences between situations. Statistically, variance restrictions may have contributed to the lack of correlation. The present non-clinical and sociodemographically homogeneous sample had a relatively low average borderline accentuation. The EMA items, additionally, referred to the period since the last survey. Responding to the items could have depended on whether individuals interacted with others during that period and how positively or negatively those interactions were perceived. Regardless of the social context, the mentalization items had to be completed during each survey.

Hypothesis 2 that lower self-certainty is associated with higher borderline accentuation could be confirmed, as we found a negative, moderate semi-partial correlation between self-certainty and borderline traits (*r*_sp_ = –0.36). Individuals with lower certainty about their own mental states showed stronger BPD-related traits, such as intense emotions and an unstable self-image. This aligns with the assumption that low self-certainty reflects hypomentalizing [36], which manifests as uncertainty about one’s feelings, thoughts, and behavior. In contrast, a coherent sense of self is associated with self-oriented mentalizing [64]. The PSDI items used also address aspects of an unstable self-image, such as fluctuating self-esteem and feelings of inner emptiness. The greater intraindividual variability in self-certainty observed in individuals with higher BPD traits (hypothesis 1) may contribute to fluctuations in self-worth, as shown in previous EMA studies [e.g., 65].

Hypothesis 3 that higher other-certainty is associated with BPD features and hypothesis 4 that higher BPD features are associated with a greater discrepancy between other- and self-certainty could both not be confirmed. One possible explanation is that other-certainty may play only a minor role in BPD, as no significant associations were found when controlling for self-certainty. Similarly, Müller et al. [36] reported mixed findings, with no correlations between other-certainty and certain psychopathological variables such as disinhibition (e.g., impulsive behavior) or interpersonal functioning, e.g., lack of ability to form stable relationships) — both central aspects of BPD [66, 67]. This suggests that while other-certainty may be related to certain forms of psychopathology, it might not be specifically linked to BPD traits. The lack of an association between borderline traits and other-self differences (hypothesis 4) suggests that a mentalizing profile with high other-certainty approaching or even exceeding self-certainty may not be maladaptive and thus not an indicator of hypermentalizing. Previous associations between BPD and hypermentalizing were mostly based on lab-based tasks like the *Movie for the Assessment of Social Cognition* (MASC) of Dziobek et al. [68], with mixed findings. This may reflect a lack of attention to real-life mentalizing dynamics. Since meta-analyses have pointed to a potential link [e.g., 69], alternative explanations should be considered for the absence of associations in EMA-based data. Steinberg et al. [27] found that borderline traits predicted within-person fluctuations in mentalizing, but not overall average levels. They suggested that EMA captures a unique aspect of mentalizing — namely, its instability — that static questionnaires may miss. In the present study, borderline traits were assessed once in the lab using the PSDI. However, both Hopwood [70] and various EMA studies [71–73] emphasize the situational and dynamic nature of personality features such as affective and interpersonal instability in BPD. The MSSDs and the moderate correlations between EMA and baseline values of other-certainty further suggest that EMA captures a distinct, real-life dimension. This is in line with EMA literature since EMA and questionnaires are conceived to assess different aspects of self-reports differentiating between the “experiencing self” (assessed in EMA) and the “remembering” and “believing” selves (measured through retrospective and trait questionnaires) [74]. This raises the question of whether single-timepoint lab assessments can adequately reflect this everyday variability. Future studies should therefore assess BPD features over time. Additionnally it should be considered what aspects of mentalizing is actually assessed by the EMA items used in this study. The study used items psychometrically evaluated in Höller et al. [37]. Even though the item set shows excellent reliability, it might not capture mentalizing as a balanced multidimensional construct. However, next to the study of Steinman et al. [27], this is the only validated measure for mentalizing in EMA research so far. Future studies should build on these findings and investigate different aspects and components of mentalizing.

### Practical implications

These findings from a non-clinical community sample suggest that low self-reported ability to recognize and interpret one’s own mental states—low self-certainty—may play a role in BPD related impairments, alongside hypermentalizing. It could be that individuals with low self-certainty might struggle with an unstable sense of self or identity confusion due to limited awareness of their intentions, beliefs, and desires. However, this must be further investigated in a sample with more BPD features or even a clinical sample. Targeting self-certainty in therapy and helping patients identify and reflect on their mental states might be one possible approach to reduce symptoms. This idea aligns with the goals of Mentalization-Based Treatment (MBT), which aims to stabilize mentalizing and the self-concept, especially within the therapeutic relationship [16]. Therapists are encouraged to promote slow, reflective mentalizing [18]. The current findings give first insights on the importance of directing patients’ attention toward their own mental states. In this non-clinical sample, self-certainty was significantly higher than other-certainty in EMA, suggesting a potentially adaptive profile. Together with its negative association with borderline traits, future research should prioritize self-certainty and investigate it further a resilience factor and thereby maybe as a promising target for early interventions.

Through Ecological Momentary Interventions [EMIs; 75], patients could receive smartphone-based prompts that guide attention toward their own mental states. Therapy could focus on stabilizing mentalizing capacities to reduce situational fluctuations. The lack of association between intraindividual fluctuations in other-certainty and BPD does not mean such variability is irrelevant in BPD. It just suggests that individuals with elevated borderline features may not necessarily exhibit greater instability in perceiving others’ mental states.

### Strength and limitations

This study was the first to examine self-reported self- and other-certainty in everyday life using EMA. The high temporal resolution provided new insights into intraindividual fluctuations in mentalizing ability. Despite using only two items per scale, reliability was acceptable and participant compliance was high. However, it remains open how well these time-based EMA items capture everyday mentalizing processes, which is particularly relevant to other-certainty. Future studies should investigate and include interpersonal context. One possible methodological approach would be to include event-based assessments following an interpersonal interaction as well as the evaluation of this interaction or audio recordings following the approach of Steinberg et al. [27]. This would ensure a more assessment of other-certainty in daily life.

Limitations include exclusive reliance on self-report measures. As it is well known, self-report measures can be biased by multiple factors, e.g., social context when assessing other-certainty, and methodological constraints. The sample was small, homogeneous (mostly young female students), and non-clinical, limiting generalizability and possibly contributing to weak effects. The biggest limitation was the use of the PSDI that may not have been ideal to assess borderline accentuation. Even though the PSDI is a non-pathological measure that relates to the non-pathological equivalents of the personality disorders of the DSM-5 [76] and ICD-10 [77] and has been used in other studies [e.g., 78–80], it is not a very commonly used questionnaire in borderline research. Future studies should therefore rely on a clinical measures for BPD features. Overall, these results offer preliminary insights into aspects of everyday mentalizing processes and their relation to borderline personality traits but must be further investigated with measures that cover more aspects of mentalizing (which still need to be developed) in a clinical sample.

## Conclusion

This study aimed to replicate prior findings on fluctuations in mentalizing and their link to borderline traits using brief EMA self-reports over one week. Results show high variability in self- and other-certainty, with self-certainty negatively predicting borderline features and its fluctuations positively associated with borderline style. Although clinical replication is needed, these findings highlight the importance of focusing on self-related mentalizing in BPD therapy and prevention. Future large-sample EMA studies are needed to confirm these results and inform targeted interventions.

## Data Availability

The dataset supporting the conclusions of this article is available in the OSF repository, 10.17605/OSF.IO/NWG89.

## Abbreviations

BPD: Borderline Personality Disorder
MBT: Mentalization-based therapy
EMA: Ecological Momentary Assessment
RFS: Reflective Functioning Scale
CAMSQ: Certainty About Mental States Questionnaire
ICC: Intraclass Correlation
